# Does Characterizing Global Running Pattern Help to Prescribe Individualized Strength Training in Recreational Runners?

**DOI:** 10.3389/fphys.2021.631637

**Published:** 2021-03-17

**Authors:** Aurélien Patoz, Bastiaan Breine, Adrien Thouvenot, Laurent Mourot, Cyrille Gindre, Thibault Lussiana

**Affiliations:** ^1^Institute of Sport Sciences, University of Lausanne, Lausanne, Switzerland; ^2^Research and Development Department, Volodalen Swiss Sportlab, Aigle, Switzerland; ^3^Department of Movement and Sports Sciences, Ghent University, Ghent, Belgium; ^4^Research and Development Department, Volodalen, Chavéria, France; ^5^Research Unit EA3920 Prognostic Markers and Regulatory Factors of Cardiovascular Diseases and Exercise Performance, Health, Innovation Platform, University of Bourgogne Franche-Comté, Besançon, France; ^6^Division for Physical Education, Tomsk Polytechnic University, Tomsk, Russia

**Keywords:** running, plyometric training, dynamic weight training, concurrent training, sports biomechanics

## Abstract

This study aimed to determine if concurrent endurance and strength training that matches the global running pattern would be more effective in increasing running economy (RE) than non-matched training. The global running pattern of 37 recreational runners was determined using the Volodalen^®^ method as being aerial (AER) or terrestrial (TER). Strength training consisted of endurance running training and either plyometric (PLY) or dynamic weight training (DWT). Runners were randomly assigned to a matched (*n* = 18; DWT for TER, PLY for AER) or non-matched (*n* = 19; DWT for AER, PLY for TER) 8 weeks concurrent training program. RE, maximal oxygen uptake V̇O_2_max) and peak treadmill speed at V̇O_2_max (PTS) were measured before and after the training intervention. None of the tested performance related variables depicted a significant group effect or interaction effect between training and grouping (*p* ≥ 0.436). However, a significant increase in RE, V̇O_2_max, and PTS (*p* ≤ 0.003) was found after the training intervention. No difference in number of responders between matched and non-matched groups was observed for any of the performance related variables (*p* ≥ 0.248). In recreational runners, prescribing PLT or DWT according to the global running pattern of individuals, in addition to endurance training, did not lead to greater improvements in RE.

## Introduction

Running economy (RE), which refers to the steady-state of oxygen consumption at a given running speed, is a critical factor of running performance (Conley and Krahenbuhl, 1980). RE improves after years of endurance running training, and especially if high volume, high intensity interval, or uphill running training are undertaken (Barnes and Kilding, 2014). Beyond running, different training strategies have been shown to potentially improve RE (Mikkola et al., 2007; Taipale et al., 2013; Barnes and Kilding, 2014). Among them, concurrent training, i.e., the use of strength training such as plyometric training (PLT) or dynamic weight training (DWT) in parallel with endurance running training, has been shown to further benefit RE (Barnes and Kilding, 2014). For instance, several studies that used this concurrent training method reported an improvement of RE ranging from 0 to 4.7% (Pellegrino et al., 2016; Meszler et al., 2019). However, the exact mechanisms leading to an improvement of RE after PLT or DWT remained unclear (Trowell et al., 2020).

PLT involves eccentric-concentric contraction cycles to allow the muscle-tendon unit to efficiently store and release elastic energy. During such cycle, there is a focus on a short ground contact time (*t*_*c*_) and a high leg stiffness (*k*_leg_) (Anderson, 1996). Common PLT exercises for runners are repeated rebound jumps or drop jumps. On the other hand, DWT involves a greater focus on concentric contractions aiming to produce a maximal power output, which is a compromise between speed and force generation (Kawamori and Haff, 2004). Common DWT exercises are, e.g., squats jumps and dynamic lunges. From a kinematic point of view, PLT implies shorter *t*_*c*_ than DWT, de facto theoretically more in line with the mechanical demands of running. Indeed, running is characterized by a short contact phase (dependent on the running speed but generally smaller than 400 ms) followed by a flight phase. Therefore, the running pattern is a succession of plyometric contractions showing a spring like behavior, as suggested by the spring mass model (Blickhan, 1989). However, all runners do not share a running pattern that equally resembles to a spring.

Indeed, some runners were shown to exhibit a more asymmetric contact phase (i.e., a longer duration of the propulsion phase than the braking phase) and less vertical oscillation of their center of mass during the flight time (*t*_*f*_) than would be predicted by the spring-mass model (Lussiana et al., 2019). Thus, these running patterns are less accurately modeled by the spring-mass model. Following such ideas, it has been show that individuals could be classified into two categories termed aerial (AER) and terrestrial (TER) using the subjective Volodalen^®^ scale (Gindre et al., 2015). Shorter *t*_*c*_ and greater *k*_leg_ are exhibited in AER than TER, while greater leg compression during stance is observed in TER compared to AER (Gindre et al., 2015). These kinematic differences might indicate that theoretically certain training modalities such as PLT or DWT might better suit AER or TER, respectively.

It is well established that individual differences exist in response to training, where *high responders* show large responses whereas *low responders* show small responses or no responses at all (Mann et al., 2014). Interestingly, Hautala et al. (2006) reported that low responders to an endurance based training program could become high responders to a strength based training program. Unfortunately, this variability in training responsiveness is not well understood and might be attributable to various factors including the absence of definition for high and low responders in the scientific literature and a one size fits all approach to exercise prescription (Mann et al., 2014). It has been purported that a more “personalized approach” to exercise prescription based on factors such as genotype, baseline phenotype, pre-training autonomic activity, individual homeostatic stress responses, recovery, and nutrition should improve training responsiveness (Mann et al., 2014). However, more research is still needed to clarify and quantify the role of these parameters. Also, for coaches, these factors are often hard to assess. In line with this view, Gindre et al. (2015) made the assumption that AER and TER could respond preferentially to different types of training interventions to improve RE. In other words, the knowledge of the global running pattern might provide useful indications for the prescription of training modalities toward an improvement in RE.

Hence, the purpose of the present study was to verify the effectiveness (i.e., mean increase in RE) and responsiveness (i.e., number of participants with a significant increase in RE) of two strength training modalities (i.e., PLT and DWT) on top of a standard endurance running training program to improve RE in runners with different global running patterns (i.e., AER and TER). We hypothesized that a training program that matches the underlying kinematics of the global running pattern (i.e., PLT for AER and DWT for TER) would be more efficient and thus would trigger a greater increase in RE and a lower rate of low-responders than a non-matched training program.

## Materials and Methods

### Participants

The study has been conducted over a 3 months period, which permitted to test 37 recreational and regular runners among which there were 5 females (age: 29.0 ± 9.0 years, height: 168 ± 6 cm, body mass: 59.3 ± 3.0 kg, weekly training hours: 2.0 ± 1.2 h) and 32 males (age: 29.4 ± 9.3 years, height: 177 ± 8 cm, body mass: 73.4 ± 12.4 kg, weekly training hours: 2.6 ± 1.3 h). For study inclusion, voluntary participants were required to be in good self-reported general health with no current or recent (<3 months) musculoskeletal injuries, and to have not previously undertaken any structured PLT or DWT. Two groups of runners were set up. The matched group consisted of AER following PLT and TER following DWT (*n* = 18). The non-matched group consisted of AER following DWT and TER following PLT (*n* = 19). As assessed by two-tailed non-matched *t*-tests, there were no significant differences in age, height, body mass, and weekly training hours between both groups (Table 1).

**TABLE 1:** Mean ± *SD* of baseline participant characteristics for matched and non-matched groups.

	**Matched (15 men, 3 women)**	**Non-matched (17 men, 2 women)**	***p***
Age (y)	30.8 ± 8.4	28.0 ± 9.8	0.350
Height (cm)	177 ± 8	175 ± 8	0.499
Body mass (kg)	72.2 ± 10.7	70.8 ± 14.3	0.730
Weekly training hours (h⋅week^–1^)	2.50 ± 1.25	2.55 ± 1.31	0.901

*No significant differences (p ≤ 0.05) were reported between both groups.*

Participants were informed of the benefits and risks of the investigation prior to signing an institutionally approved informed consent document to participate in the study. They were informed that the data and results were confidential, and that they could withdraw at any time during the study, that was approved by an Institutional Review Board of the University of Bourgogne, Franche-Comté (CPP: 2014-A00336-41) and adhered to the latest Declaration of Helsinki of the World Medical Association (World Medical Association, 2013).

### Experimental Approach to the Problem

After providing written informed consent, participants performed an initial baseline experimental session including a series of tests. These tests consisted of the assessment of the global running pattern using the Volodalen^®^ scale to classify a runner as AER or TER, jump tests to evaluate the explosive concentric capacity and plyometric characteristics of the lower limb, a submaximal running test to determine RE, and a maximal incremental running test to determine peak treadmill speed (PTS) and maximal oxygen uptake ($\dot{\text{V}}\,{\text{O}}_{2}$max) (Gindre et al., 2015). Tests were interspersed by a 5-min passive recovery in a seated position. After that, each participant was randomly assigned to one of two 8 week concurrent training modalities, i.e., a standard endurance running training program combined with either PLT or DWT. After this assignment, participants were regrouped for statistical analysis based on whether their running pattern (AER or TER) was matched or non-matched with their prescribed strength training (DWT or PLT).

### Procedures

#### Global Running Pattern Assessment

During the warm-up of the initial baseline experimental session (5 min on a treadmill at 9 km⋅h^–1^), two running coaches with more than 3 years of experience using the Volodalen^®^ method (CG and TL) paid attention to five key elements: vertical oscillation of the head, antero-posterior motion of the elbows, pelvis position at ground contact, foot position at ground contact, and foot strike pattern. Each element was scored from one to five, leading to a global subjective score (V^®^ score) that represents the global running pattern of participants. This score ultimately allows the classification of runners into the two different categories (i.e., AER if V^®^ score > 15 and TER otherwise). The Volodalen^®^ method was fully described and studied elsewhere (Gindre et al., 2015) and was shown to be a reliable method to assess running pattern (Patoz et al., 2019). The two coaches disagreed in their assessment of 3 individuals (8.1%). In these cases, the two coaches adopted a consensus following a discussion.

#### Endurance Running Training

All participants followed a basic endurance running training in line with what they were used to do before the study. Noteworthy, participants were not following a proper periodization training before starting the given training, i.e., they were not in a specific phase of a global periodization training. Training was divided into three different intensities based on their PTS: below 80%, between 80 and 95%, and between 95 and 105% of PTS. These percentages were chosen as to represent an aerobic, threshold and high intensity zone, respectively. The prescribed time in each of these three training zones during the 8 weeks training is described in Table 2. Main training volume (70–80%) was spent at running speeds below 80% of PTS.

**TABLE 2:** Characteristics of the 8 weeks training program.

**Weeks**	**1**	**2**	**3**	**4**	**5**	**6**	**7**	**8**
**Endurance training**								
Volume (min)	130	135	145	150	160	165	170	175
Intensity < 80% PTS (min)	104 (80%)	106 (79%)	113 (78%)	114 (76%)	121 (76%)	121 (73%)	122 (72%)	123 (70%)
80% < Intensity < 95% PTS (min)	17 (13%)	19 (14%)	21 (14%)	24 (16%)	27 (17%)	30 (18%)	33 (19%)	35 (20%)
95% < Intensity < 105% PTS (min)	9 (7%)	10 (7%)	11 (8%)	12 (8%)	13 (7%)	14 (9%)	16 (9%)	17 (10%)
**Strength training (PLT and DWT)**								
Volume (min)	40	40	62	62	62	62	80	80
Session * cycle (per week)	1 * 4	1 * 4	1 * 4 + 1 * 2	2 * 4 + 1 * 2	3 * 4 + 1 * 2	4 * 4 + 1 * 2	2 * 4	2 * 4
Warm up (min)	7	7	7	7	7	7	7	7
Time per exercise (sec)	20	25	30	30	35	35	40	40
Rest between exercise (sec)	40	35	30	30	25	25	20	20
Rest between cycle (min)	3	3	3	3	3	3	3	3

*PTS, Peak treadmill speed; PLT, plyometric training; DWT, dynamic weight training.*

Basic endurance sessions consisted of continuous running for 45–75 min, predominantly at a running speed below 80% of PTS with some unstructured bouts of faster running at 80–95% of PTS between 10 and 25 min per session. Interval sessions consisted of a 15 min easy warm-up at a running speed below 80% of PTS and involved repeated interval bouts ranging from 30 s to 2 min at 95–105% of PTS for an accumulated total of 6–12 min of fast running per session. In the beginning of the 8 weeks training plan, an example interval session consisted of 2 times 6 min of (30 s at 100% of PTS—30 s below 80% of PTS) with 2 min recovery between each 6 min block while at the end of the 8 weeks training plan, an example interval session consisted of 3 blocks of 2 repetitions of (2 min at 100% of PTS—1 min 30 s below 80% of PTS) with 5 min recovery between each block.

#### Plyometric or Dynamic Weight Training

**FIGURE 1:**
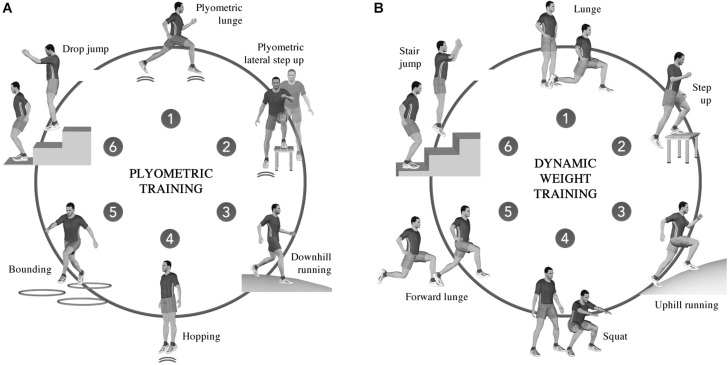
Circuit training protocol for the plyometric training **(A)** and dynamic weight training **(B)**.

Participants were asked to perform a predetermined circuit training composed of six exercises and designed as PLT or DWT (Figure 1). Details of the 8 weeks training are given in Table 2. Participants performed the same circuit training during the entire protocol but with progressive changes in the number of cycles and the exercise/rest ratio. Hence, despite different exercises, the total training load (total duration of effort and resting periods) was aimed to be equivalent between groups. Also, as the participants had no previous experience in resistance training, only body weight was used.

#### Jump Test

The squat jump test (SJ) was used to evaluate the explosive concentric capacity of the lower limbs (Bosco et al., 1983). Participants were required to jump vertically as high as possible from a static squat position and to start the landing with knees straight and ankles plantar-flexed. The depth of the squat was self-selected. Participants had to maintain the static squat position for two seconds prior to the jump. Squat jump height (SJ-h, in cm) was calculated from flight time (*t*_*f*_) (Eq. 1) as measured by an optical measurement system (Optojump Next^®^, MicroGate Timing and Sport, Bolzano, Italy) sampling at 1,000 Hz.

(1) $$\mathrm{SJ}-h=\frac{g\,t_{f}^{2}}{2}$$

Following the SJ, a five-repetition rebound jump test (5RJ) was used to evaluate the plyometric characteristics of the participants’ lower limbs (Bosco et al., 1983; Dalleau et al., 2004). Participants were required to jump vertically as high as possible while minimizing ground contact time *(t_*c*_)* and maximizing flight time *(t_*f*_)*. Participants were also instructed to minimize knee actions (i.e., flexion and extension) during the test. *t*_*f*_ and *t*_*c*_ were measured by the Optojump Next^®^ system. The average mechanical power during the positive (concentric) work per body mass (5RJ-P, in W⋅kg^–1^) was then calculated on the basis of the methods described by Bosco et al. (1983) using the following formula (Eq. 2).

(2) $$5\,R\,J-P=\frac{g^{2}\,t_{f}\,\left(t_{c}+t_{f}\right)}{4\,t_{c}}$$

All jumps were performed with hands placed on the hips and participants were wearing their habitual running shoes. After five practice trials of each jump, three repetitions of each jump test were performed with a 30 s rest between repetitions and a 2 min rest between the SJ and 5RJ tests. The best repetition of the SJ (based on the longest *t*_*f*_) and 5RJ (based on the highest average mechanical power) was used for statistical analysis.

#### Submaximal Running Test

Participants ran for 5 min on a treadmill at 12 km⋅h^–1^. Gas exchange was measured breath-by-breath using a gas analyser (Cortex Metamax 3B, Cortex Biophysik, Leipzig, Germany) and subsequently averaged over 10 s intervals throughout the test. Before each test, the gas analyzer was calibrated following the manufacturer’s recommendations using ambient air (O_2_: 20.93% and CO_2_: 0.03%) and a gas mixture of known composition (O_2_: 15.00% and CO_2_: 5.00%). The spirometer was calibrated using a 3 L syringe. Respiratory exchange ratio (RER), oxygen uptake (V̇O_2_), and carbon dioxide output (V̇O_2_) were averaged over the last minute of the 5 min running trial. RER had to remain below 1.0 during the trials for the data to be included in the analysis, otherwise the corresponding data were excluded as deemed to not represent a submaximal effort. In such case, the selected submaximal speed was lowered iteratively by 1 km⋅h^–1^ until an RER below 1.0 was achieved. This resulted in submaximal testing speeds of 9 (*n* = 1), 10 (*n* = 6), 11 (*n* = 5), and 12 km⋅h^–1^ (*n* = 25). These speeds were kept the same for the post testing. RE was calculated from the running velocity divided by net V̇O_2_ normalized to individual body mass (m⋅ml^–1^⋅kg^–1^) where net V̇O_2_ = V̇O_2_−restV̇O_2_, with rest V̇O_2_ given by the average over the last minute of a 5 min upright stance measure prior to the submaximal running test. This choice of units for RE have a conceptual advantage that numerical values are directly related to RE (i.e., the larger the numerical value, the better the RE) (Turner et al., 2003).

#### Maximal Incremental Test

Following the submaximal test, participants performed a maximal incremental running test on the treadmill. Starting at 8 km⋅h^–1^, the treadmill speed was increased by 0.5 km⋅h^–1^ every minute until volitional exhaustion. The participants received strong verbal encouragement to ensure attainment of maximal values during the test. $\dot{\text{V}}\,{\text{O}}_{2}$max, averaged over 30 s, was said attained when two or more of the following criteria were met: an increase in $\dot{\text{V}}\,{\text{O}}_{2}$ less than 2.1 ml⋅kg^–1^⋅min^–1^ between two consecutive stages, an RER greater than 1.1, and a heart rate (RS810, Polar Electro Oy, Kempele, Finland) of ±10 beats per minute of the predicted maximal heart rate value (i.e., 220−age), as done by Howley et al. (1995). PTS is defined as the running speed of the last fully completed increment (MAS) plus the fraction of time spent in the following uncompleted increment (α) multiplied by the running speed increment (*Δs* = 0.5 km⋅h^–1^) (Kuipers et al., 2003):PTSMAS + α△*s*.

### Statistical Analyses

Assuming a medium effect size [partial eta squared (η^2^*_*p*_*) = 0.06] in RE improvement between matched and non-matched training groups, an α error of 0.05, and a power of 0.8, sample size calculations resulted in the requirement of 34 participants (Faul et al., 2007). However, the 37 participants were kept to slightly increase statistical power. Test-retest reliability coefficients ranged from *r* = 0.805 to 0.954 (*p* < 0.001) indicating good to excellent reliability of measurements. Descriptive statistics are presented using mean ± standard deviation. Effect sizes are reported as η^2^*_*p*_* values. The normality of the data and homogeneity of variances were verified using Shapiro-Wilk (*p* range: 0.163–0.943) and Levene’s test (*p* range: 0.162–0.880), respectively. Unpaired two-sided Student’s *t*-tests were used to compare participant characteristics between matched and non-matched groups at baseline. Statistical analysis was performed using Jamovi [version 1.0.8 (Computer Software), retrieved from https://www.jamovi.org] with a level of significance set at *p* ≤ 0.05.

#### Effectiveness of the Training Interventions

A pre-post experimental design was used with two training groups (matched vs. non-matched). Effectiveness of the training protocol on performance parameters (primary criteria RE; secondary criteriaV̇O_2_max, PTS, SJ-h, and 5RJ-P) was assessed by repeated measures ANOVA (RM-ANOVA) with pre vs. post testing as within-subject factor and matched vs. non-matched grouping as between subject factor, and employing Bonferroni procedures for pair-wise *post-hoc* comparisons.

#### High Responders vs. Low Responders

Participants were all labeled as a responder or non-responder for the three performance variables that were significantly influenced (significant pre-post effect reported by the RM-ANOVA) by the protocol (i.e., RE, $\dot{\text{V}}\,{\text{O}}_{2}$max, and PTS) based on set % changes derived from the literature. A participant was determined as responder when RE increased by more than 2.6% (Barnes and Kilding, 2015), $\dot{\text{V}}\,{\text{O}}_{2}$max by more than 5.9% (Dalleck et al., 2016), and PTS by more than 4% (arbitrary cut-off). Chi-squared analyses (χ^2^) were performed on the number of responders and non-responders to assess if there was a difference of responsiveness for any of the three performance variables within matched and non-matched groups.

## Results

### Effectiveness

No significant group effect or interaction effect were found between the training (pre-post) and grouping (matched vs. non-matched) for any of the tested performance related variables. These results indicate that the effect of the applied training intervention on the performance related variables did not significantly differ between the matched and non-matched groups (*p* ≥ 0.436; Table 3). However, we found a significant increase in RE, PTS, and $\dot{\text{V}}\,{\text{O}}_{2}$max after the training intervention (*p* ≤ 0.003; Table 3). Noteworthy, *post-hoc* comparisons were not investigated as no interaction effect was reported.

**TABLE 3:** Mean ± *SD* for running economy (RE), maximal oxygen uptake ($\dot{\text{V}}\,{\text{O}}_{2}$max), peak treadmill speed (PTS), squat jump height (SJ-h), and average mechanical power during the positive (concentric) work per body mass of five repeated rebound jumps (5RJ-P) per training group, pre and post the training intervention as well as main effects (pre-post and group) and interaction effect (pre-post × group) for these five performance related variables.

		**Pre**	**Post**
**RE (m⋅ml^–1^⋅kg^–1^)**	**Matched**	5.09 ± 0.44	5.18 ± 0.53
	**Non-matched**	5.18 ± 0.35	5.32 ± 0.39
	Main effect group	*p* = 0.398	η^2^*_*p*_* = 0.020
	Main effect pre-post	***p* = 0.003**	**η^2^*_*p*_* = 0.223**
	Interaction pre-post x group	*p* = 0.565	η^2^*_*p*_* = 0.010
$\dot{\text{V}}\,{\text{O}}_{2}$**max (ml⋅min^–1^⋅kg^–1^)**	**Matched**	53.4 ± 8.27	55.6 ± 7.35
	**Non-matched**	54.9 ± 8.14	56.3 ± 7.54
	Main effect group	*p* = 0.663	η^2^*_*p*_* = 0.005
	Main effect pre-post	***p* = 0.002**	**η^2^*_*p*_* = 0.244**
	Interaction pre-post x group	*p* = 0.465	η^2^*_*p*_* = 0.015
**PTS (km⋅h^–1^)**	**Matched**	15.1 ± 1.83	15.8 ± 1.67
	**Non-matched**	15.7 ± 1.63	16.2 ± 1.48
	Main effect group	*p* = 0.353	η^2^*_*p*_* = 0.025
	Main effect pre-post	***p* < 0.001**	**η^2^*_*p*_* = 0.598**
	Interaction pre-post x group	*p* = 0.436	η^2^*_*p*_* = 0.017
**SJ-h (cm)**	**Matched**	30.9 ± 5.3	31.8 ± 5.4
	**Non-matched**	31.0 ± 5.6	31.7 ± 5.1
	Main effect group	*p* = 0.996	η^2^*_*p*_* = 0.000
	Main effect pre-post	*p* = 0.064	η^2^*_*p*_* = 0.095
	Interaction pre-post x group	*p* = 0.888	η^2^*_*p*_* = 0.001
**5RJ-P (W)**	**Matched**	35.7 ± 6.6	36.0 ± 7.6
	**Non-matched**	36.6 ± 7.9	37.1 ± 5.7
	Main effect group	*p* = 0.647	η^2^*_*p*_* = 0.006
	Main effect pre-post	*p* = 0.606	η^2^*_*p*_* = 0.008
	Interaction pre-post × group	*p* = 0.952	η^2^*_*p*_* = 0.000

*Significant effects (p ≤ 0.05) are reported in bold font. Effect size is reported as partial eta squared (η^2^_*p*_) values.*

### Responsiveness to Training

**FIGURE 2:**
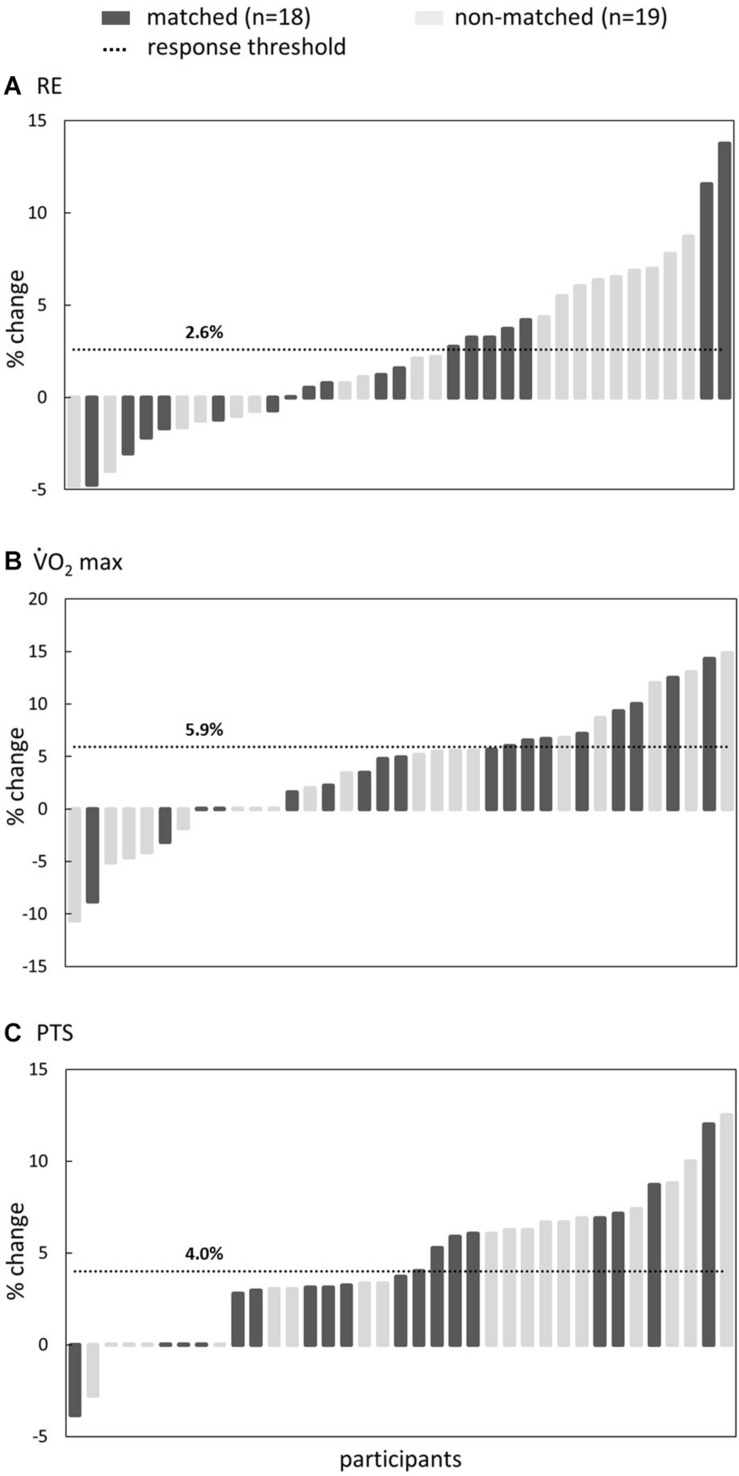
Individual response (in % change) for **(A)** running economy (RE), **(B)** maximal oxygen uptake ($\dot{\text{V}}\,{\text{O}}_{2}$max), and **(C)** peak treadmill speed (PTS). Dark gray and light gray bars indicate the matched and non-matched groups, respectively. The horizontal line represents the threshold for identification of a participant as a (non-)responder. Participants are ranked for each variable from least (left) to most (right) desired effect.

No statistical difference in the responsiveness to training intervention were found between matched and non-matched groups for any of the performance related variables that were significantly influenced (significant pre-post effect reported by the RM-ANOVA; Table 3) by the protocol (*p* ≥ 0.248; Table 4). Individual responses are shown in Figure 2.

**TABLE 4:** Results of Chi-squared (χ^2^) tests on the number of responders and non-responders for running economy (RE), maximal oxygen uptake ($\dot{\text{V}}\,{\text{O}}_{2}$max), and peak treadmill speed (PTS) within the matched and non-matched training groups.

	**Matched**		**Non-matched**			
	**Responder**	**Non-responder**	**Responder**	**Non-responder**	**χ^2^**	***p***
RE	7	11	9	10	0.271	0.603
$\dot{\text{V}}\,{\text{O}}_{2}$max	8	10	5	14	1.33	0.248
PTS	8	10	9	10	0.032	0.858

*No significant difference (p ≤ 0.05) was observed.*

## Discussion

This study aimed at determining the effectiveness and responsiveness to two strength training modalities (i.e., PLT and DWT) combined with standard endurance training to improve RE in recreational runners. Identifying the global running pattern (i.e., TER or AER) and matching it to a PLT or DWT type of strength training prescription resulted in similar RE improvements and response to training than if no matching had been performed. As such, the results of the present study could not support our hypotheses. The following discussion is elaborating on possible explanations for the rejection of our hypothesis and directions for future research.

When following a certain training intervention, some individuals show a large positive response while others a small or even no response (Mann et al., 2014). Moreover, individual responsiveness to training can vary by training mode (Hautala et al., 2006). Likewise, in the present study we aimed at improving RE and found a significant increase of 2.3% at groups level, but with individual effect ranging from a 4.8% decrease to a 13.8% increase. This was concomitant with an average PTS increase of 4.3% (ranging from a 3.8% decrease to a 12.5% increase) and a $\dot{\text{V}}\,{\text{O}}_{2}$max increase of 3.8% (ranging from a 10.6% decrease to a 14.8% increase). This wide range of individual responses highlights the importance of taking into account individual responses and not only the training effect on a group level (see Figure 2). It has been reported that average group level increases in RE ranged from 0 to 4.7% using heavy weight strength training (Johnston et al., 1997) and 0–4% using explosive training (Pellegrino et al., 2016; Meszler et al., 2019), which is in line with the current findings. Nevertheless, several studies reported no significant increase in RE following concurrent endurance and strength training in recreational athletes despite improvements in muscle strength (Ferrauti et al., 2010; Mikkola et al., 2011; Taipale et al., 2013; Damasceno et al., 2015).

A possible explanation for the relatively small improvements in RE in our study can be found in the duration of the training period. A review by Denadai et al. (2017) highlighted that longer training periods (>8–21 weeks) are likely to result in greater RE improvements than shorter programs (6–8 weeks) due to the time-course of neuromuscular adaptations following concurrent training. A review on this topic by Rønnestad and Mujika (2014) stated that the possible mechanisms of how concurrent endurance and strength training can improve RE are related to a delayed activation of less efficient type II muscle fibers, an improved neuromuscular efficiency, the conversion of fast-twitch type IIx fibers into more fatigue resisted type IIa fibers, and an improved musculo-tendinous stiffness. During a short to medium term training period (up to 8 weeks), as was the case in the current study, the expected neuromuscular adaptations are an increased neural activation and a smaller relative proportion of type IIx than type IIa fibers, while an optimized musculo-tendinous stiffness is only achieved after longer training periods (>8–21 weeks). The absence of significant effects on any of the jumping performance parameters (SJ-h and 5RJ-P) in this study confirms a possible lack of musculo-tendinous adaptations after the 8 weeks training period. Therefore, when aiming at improving RE by strength training, longer training periods (>8–21 weeks) are advised.

Another possible explanation for unachieved RE improvement through concurrent training in some studies is that because the exact mechanisms behind RE improvement are still unknown, or the appropriate stimulus is not used. Some studies were able to induce neuromuscular adaptations (i.e., increase in maximal and explosive strength) through heavy weight training, while the intended effects on RE remained absent (Taipale et al., 2013; Vikmoen et al., 2016). A recent review on the effect of strength training on biomechanical and neuromuscular adaptations concluded that evidences that neuromuscular effects obtained by strength training transfer to running biomechanics are lacking (Trowell et al., 2020). In this study we tried to match the concurrent strength training to the global running pattern in order to obtain individualized neuromuscular adaptation in an attempt to maximize RE improvements. However, as we did not obtain better results, it seems that more insights are needed regarding the interaction between neuromuscular stimuli, its resulting adaptations, their transfer to the running pattern and their impact on RE.

A possible limitation to the current study might be that executed training sessions were unsupervised. Indeed, after an initial supervised strength training session, athletes were instructed to perform the training sessions on their own. Moreover, as runners were novice to strength training, the load of the training sessions was kept submaximal to avoid injuries. Higher training load might lead to greater RE improvements. Therefore, the adherence, intensity, order, and organization of the training sessions was not strictly controlled and could partly explain the low mean training responses as well as the large inter-individual differences. On the other hand, it represents real-life conditions. In addition, even though runners were used to do interval trainings before starting the endurance training, the fact that they were now following a structured endurance training instead of their own “unstructured” one might partly explain the increase of $\dot{\text{V}}\,{\text{O}}_{2}$max, PTS, and RE. Finally, RE was assessed at a fixed running speed (12 km⋅h^–1^), which was obviously not individualized for each participant. An alternative could have been to determine RE at different running speeds, as long as they fall below the respiratory compensation point and that a steady-state of oxygen consumption was reached within 3–15 min (Barnes and Kilding, 2015). These speeds could have been chosen to correspond to theoretical optimal running speeds to run 5, 10, 21, and 42 km races for each individual or to participants personal best on these distances. However, obtaining these speeds would have required to perform the maximal incremental running test before the submaximal one and to perform several submaximal tests, which would have increase the duration of the overall testing.

Future research should continue to focus on how neuromuscular adaptations, induced by individualized concurrent strength and endurance training, relate to changes in running biomechanics and improvement in performance (Trowell et al., 2020). Specific attention should go to the time-course of these adaptations to reveal if improvements are mainly made during the initial training period or if more long-term progress can be made, depending on the intervention. As well, a specific evaluation of the individually different responses to strength training, including PLY and DWT, should be done. Understanding these mechanisms should help predetermining which runner (i.e., global running pattern) needs which additional strength training to optimize performance. Such an individualized approach remains the ultimate goal for coaches and athletes.

As practical guidelines we can conclude that inter individual differences in training response to concurrent training are substantial (Figure 2). We encourage coaches and athletes to regularly evaluate the effectiveness of the prescribed training program and to keep looking for ways to individualize and optimize training responses. An initial assessment of the global running pattern as being rather AER or TER using the Volodalen method can be used as a way to identify the runners’ preferences and be a guideline for training individualization.

## Conclusion

As a conclusion, prescribing PLT or DWT strength training based on global running pattern, in addition to regular endurance training, did not lead to greater improvements in RE for recreational runners. In order to be able to optimize strength training prescription and its individualization in endurance runners, future research should aim to understand the exact mechanisms relating strength training to the resulting neuromuscular and biomechanical adaptations while running.

## Data Availability Statement

The raw data supporting the conclusions of this article will be made available by the authors, without undue reservation.

## Ethics Statement

The studies involving human participants were reviewed and approved by Institutional Review Board of the University of Bourgogne, Franche-Comté (CPP: 2014-A00336-41). The patients/participants provided their written informed consent to participate in this study.

## Author Contributions

CG, LM, and TL: conceptualization, methodology, and supervision. AT and TL: investigation. AP, BB, and TL: formal analysis and writing—original draft preparation. AP, BB, AT, LM, CG, and TL: writing—review and editing. All authors contributed to the article and approved the submitted version.

## Conflict of Interest

CG was on the origin of the Volodalen^®^ method. However, this paper does not constitute endorsement of the method by the authors and stems completely from a Ph.D. research project undertaken at the Bourgogne Franche-Comté University by TL. The remaining authors declare that the research was conducted in the absence of any commercial or financial relationships that could be construed as a potential conflict of interest.
